# Amorphous silicon dioxide nanoparticles modulate immune responses in a model of allergic contact dermatitis

**DOI:** 10.1038/s41598-019-41493-7

**Published:** 2019-03-25

**Authors:** Brian C. Palmer, Samreen Jatana, Sarah J. Phelan-Dickinson, Lisa A. DeLouise

**Affiliations:** 1 Department of Environmental Medicine, University of Rochester Medical Center, New York, USA; 2 Department of Biomedical Engineering, University of Rochester, Rochester, New York USA; 3 Department of Dermatology, University of Rochester Medical Center, Rochester, New York USA

## Abstract

Amorphous silicon dioxide nanoparticles (SiNPs) are ubiquitous, and they are currently found in cosmetics, drugs, and foods. Biomedical research is also focused on using these nanoparticles as drug delivery and bio-sensing platforms. Due to the high potential for skin exposure to SiNPs, research into the effect of topical exposure on both healthy and inflammatory skin models is warranted. While we observe only minimal effects of SiNPs on healthy mouse skin, there is an immunomodulatory effect of these NPs in a model of allergic contact dermatitis. The effect appears to be mediated partly by keratinocytes and results in decreases in epidermal hyperplasia, inflammatory cytokine release, immune cell infiltration, and a subsequent reduction in skin swelling. Additional research is required to further our mechanistic understanding and to validate the extent of this immunomodulatory effect in human subjects in order to assess the potential prophylactic use of SiNPs for treating allergic skin conditions.

## Introduction

Amorphous silicon dioxide particles are typically produced via the Stöber or microemulsion method^1,2^. These particles, comprised of a matrix of alternating silicon and oxygen atoms, are negatively charged, due to the presence of silanol functional groups on the surface^3^. Either synthesis method may be used to create nanoparticles (NP), defined as particles with a single dimension of <100 nm^4^. Amorphous SiNPs are approved by the United States Food and Drug Administration (FDA) for use as anti-caking agents in foods, drugs, and cosmetics^5^. Due to the perceived safety and the wide array of surface and structural modifications available, SiNPs are currently the subject of several studies examining potential uses for drug delivery^6–8^, bio-sensing^9^, and vaccination^10–13^.

Both the increased use of SiNPs in cosmetics and the new research utilizing these NPs as transdermal drug delivery agents will lead to increased dermal exposure to SiNPs. While there are numerous studies examining the *in vivo* toxicity of high dose, acute exposures to amorphous SiNPs on the lungs^14–17^ and gastrointestinal tracts^18,19^ of rodents, there are relatively few studies examining the dermal toxicity of these NPs. Early studies of SiNP dermal toxicity revealed dose dependent cytotoxicity of *in vitro* keratinocytes and Langerhan’s cells^20,21^, associated with increases in reactive oxygen species (ROS) generation. Nabeshi *et al*. identified that 70 nm SiNPs penetrate murine skin and increase apoptosis of skin cells after 28 days of high dose (250 μg/ear/day) exposure^22^. Contrary to this early work, Ryu *et al*. found no evidence of dermal or systemic toxicity in rats exposed to daily doses (up to 2000 mg/kg) of topically applied 20 nm SiNPs for 90 days^23^. More recently, Matsuo *et al*. exposed mice to high doses (125 μg/ear/day) for 28 days and observed skin penetration of 70 nm SiNPs, but detected minimal dermal toxicity^24^. Due to the increased dermal exposure of SiNPs and the prevalence of inflammatory skin conditions^25–27^, more dermal toxicity studies are warranted to examine the effects of physiologically relevant doses of NPs on both healthy and diseased skin models.

Inflammatory skin conditions, like atopic dermatitis or psoriasis, lead to barrier defects that could potentially increase NP penetration through skin^28–31^. While literature suggests that SiNPs have minimal effects on healthy skin, little is known about interactions with inflamed skin. Our lab has previously shown that acute topical exposure to 4 μg/ear of unmodified, negatively charged amorphous SiNPs smaller than ~200 nm in diameter have an immunosuppressive effect on the 2, 4-dinitrofluorobenzene (DNFB) induced contact hypersensitivity (CHS) response in a hairless C57BL/6 mouse model^32^. The CHS response is a commonly used model of allergic contact dermatitis, consisting of both a sensitization phase and an elicitation phase^33^. After 24 hours, the SiNPs resulted in a decrease in DNFB induced ear swelling; however, the mechanism of SiNP induced dermal immunosuppression remains unknown. Here, we examine the histology, inflammatory cytokine expression, and immune cell infiltration in the skin of mice exposed to DNFB and SiNPs, to further characterize the immunosuppressive effects.

## Results

### Particle characterization of 20 nm and 400 nm silica particles

**Figure 1:**
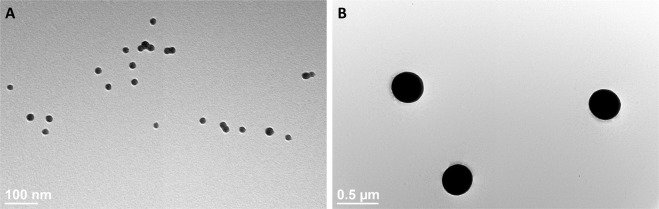
Characterization of 20 nm and 400 nm silica particles. The undiluted suspensions of the 20 nm (**A**) and 400 nm (**B**) particles were dried onto TEM grids, and representative TEM images display the size and shape of both nanoparticles.

The 20 nm SiNPs and 400 nm silica microparticles were purchased from Nanocomposix, suspended in water at concentrations of 5 and 10 mg/mL, respectively. The transmission electron microscopy (TEM) images show that both particles are homogeneous and spherical in shape (Fig. 1A,B). The hydrodynamic diameter of both the 20 nm and 400 nm particles are close to the vendor reported physical diameter of the particles and the polydispersity indices are relatively low, suggesting that each mixture is a monodisperse suspension of particles (Table 1). As previously mentioned, SiNPs contain exterior silanol groups, which create a negatively charged surface. The zeta potentials of both particles are negative, which decreases particle agglomeration by enhancing particle repulsion in suspension. The particle number and surface area for each particle are presented as they are reported from the vendor (Table 1).

**Table 1:** Characterization of 20 nm and 400 nm silica particles.

SiNPs	Hydrodynamic Diameter (nm)	Zeta Potential (mV)	Polydispersity Index	Particle Number/mL	Surface Area (m^2^/g)
20 nm	33.5 (+/− 3.3)	−21.9 (+/− 10.1)	0.236 (+/− 0.01)	4.0 × 10^14^	118.4
400 nm	438.7 (+/− 0.6)	−56.7 (+/− 0.4)	0.038 (+/− 0.02)	1.4 × 10^11^	6.7

Both particles were examined via a Malvern zetasizer to assess the hydrodynamic diameter, zeta potential, and polydispersity index.

### The 20 nm SiNPs dose dependently reduce CHS responses

**Figure 2:**
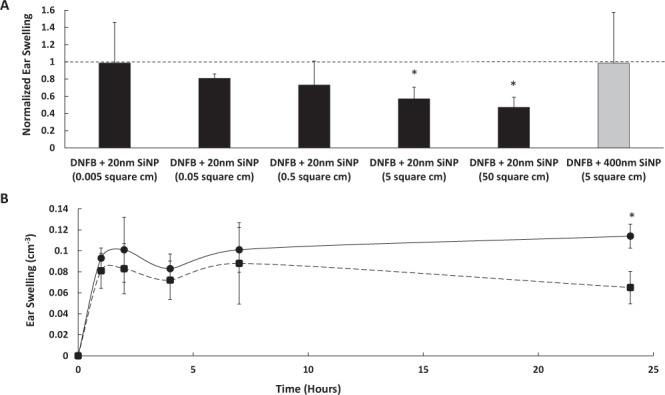
The 20 nm SiNPs dose dependently decrease DNFB induced CHS ear swelling. The effect of the SiNPs on the DNFB induced swelling response of the ears is illustrated by normalizing the average ear swelling in the NP treated left ear (bars) to the DNFB only treated right ear (dashed line). The different doses of 20 nm SiNPs are represented by the black bars, and the 400 nm silica particles dose is represented by the gray bar (**A**). The 5 cm^2^ dose of 20 nm SiNPs was used in the time course study to assess when the DNFB induced swelling response is suppressed, and a difference in ear thickness is observed between ears treated with only DNFB (solid line) and those treated with DNFB and the 20 nm SiNPs (dashed line) (**B**). The figures represent the means (SD), n = 5, and the * indicate significance compared to corresponding DNFB only treatment.

Previously, we reported that negatively charged SiNPs ≤160 nm in diameter reduced the DNFB induced swelling response in mouse ears but negatively charged 400 nm silica microparticles did not^32^. However, the NPs were applied at the same mass dose (4 μg/ear), resulting in sizable differences in surface area. When controlling for surface area (Fig. 2A), the mice displayed a dose dependent decrease in ear swelling after pretreatment with 20 nm SiNPs; reaching significance at the 5 cm^2^ and 50 cm^2^ doses, compared to ears treated with only DNFB. Importantly, the data shows that while a 5 cm^2^ dose (4 ug/ear) of 20 nm SiNPs is immunosuppressive, a 5 cm^2^ (70.7 μg/ear) dose of 400 nm silica microparticles is not, thus indicating a NP size effect. One possible mechanism of action for the NP induced immunosuppression is decreasing DNFB bioavailability by particle binding; however, the DNFB is applied to the ear in a 9,500 molar excess, relative to the 5 cm^2^ dose of SiNPs. It is unlikely that decreased DNFB bioavailability alone could cause the 50% reduction in ear swelling, and ultraviolet-visible spectroscopy studies show no significant reductions in DNFB concentrations, after incubation with 5 cm^2^ doses of 400 nm silica microparticles (Supplementary Fig. 1). The CHS data reinforces this conclusion since both the 20 nm and 400 nm silica particles were applied at the same surface area, but resulted in different levels of ear swelling reduction.

Literature suggests that CHS is a biphasic response, with both an early (1–2 hour) swelling phase mediated by the innate immune response, and a late (24 hour) phase swelling response initiated by the adaptive immune system^33–35^. To identify whether the 20 nm SiNPs inhibits one response or both, mice were pretreated with 4 μg of SiNPs on one ear and vehicle on the other. After 30 minutes, the mice were exposed to 0.2% DNFB on both ears, and they were measured at the 1, 2, 4, 7, and 24 hour time points. The 20 nm SiNPs had no effect on the ear swelling at the 1, 2, 4, or 7 hour time points; however, the swelling at 24 hours was reduced by nearly 50 percent (Fig. 2B). The preferential effect on the late phase swelling response suggests an influence on the adaptive immune response; however, the NPs must be present in the early phase of CHS (Supplementary Fig. 2), which suggests the blockade of an early signaling event that has downstream effects on ear swelling. Furthermore, while the 20 nm SiNPs decrease the ear swelling in the elicitation phase of the CHS response, they have no significant effect on the sensitization potential of DNFB, as indicated by the level of cell proliferation in the skin draining lymph nodes of DNFB sensitized mice, compared to DNFB and 20 nm SiNP co-sensitized mice (Supplementary Fig. 3). This data is consistent with our previously reported findings that quantum dots have no effect on the CHS response, when applied in the sensitization phase^32^.

### The 20 nm *S*iNPs decrease DNFB induced epidermal hyperplasia and mast cell/basophil degranulation

**Figure 3:**
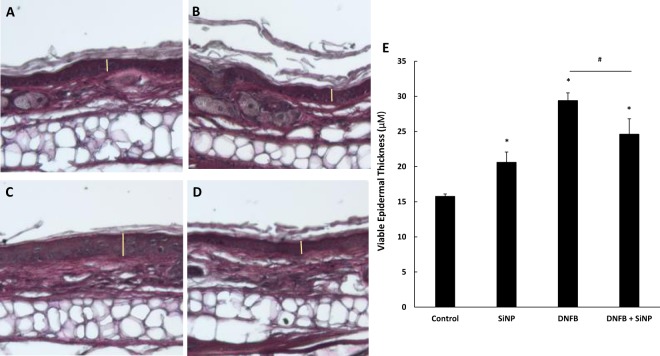
The 20 nm SiNPs decrease DNFB induced epidermal hyperplasia. The mouse ears were treated with either vehicle (**A**), 20 nm SiNPs only (**B**), DNFB only (**C**), or DNFB + 20 nm SiNPs (**D**) for 24 hours. The resulting changes in viable epidermal thickness, represented by the yellow lines in the images, are averaged in the bar graph (**E**). The graphs represent the mean (SD), n = 3, and the * indicates significance compared to control, while the # indicates significance between groups.

**Figure 4:**
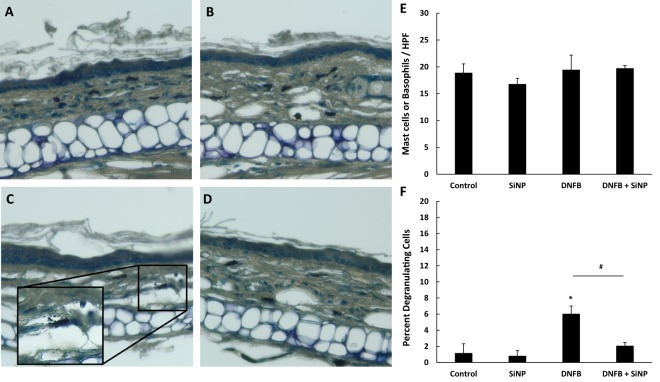
The 20 nm SiNPs decrease DNFB induced mast cell/basophil degranulation. The mouse ears were treated with either vehicle (**A**), 20 nm SiNPs only (**B**), DNFB only (**C**), or DNFB + 20 nm SiNPs (D) for 24 hours. The changes in mast cell/basophil cell number (**E**) and the percent of degranulating cells (**F**) were calculated by counting the cells in 10 high powered fields. The graphs represent the mean (SD), n = 3, and the * indicates significance compared to control, while the # indicates significance between groups.

The allergen DNFB is both a sensitizer and an irritant, and exposure to this allergen in the elicitation phase of the CHS response leads to not only swelling, but also a number of molecular and cellular changes in the skin^36^. Histological analysis was performed to assess these changes. Each mouse was treated on the ear with either vehicle, 4 μg of 20 nm SiNPs, 0.2% DNFB, or 4 μg of 20 nm SiNPs 30 minutes prior to 0.2% DNFB. After 24 hours, the ear sections were processed and stained with either a hematoxylin/eosin stain for general histology or a toluidine blue stain for mast cell and basophil enumeration. DNFB, like many irritants, is known to induce epidermal hyperplasia^37^. The sections (Fig. 3A–D) indicate that 20 nm SiNPs alone increased epidermal thickness relative to the vehicle control, indicating that SiNPs may be a low level skin irritant. Remarkably, the 20 nm SiNPs reduced the DNFB induced epidermal hyperplasia, suggesting a potential decrease in the skin irritation caused by DNFB (Fig. 3E). Skin sections were also analyzed (Fig. 4A–D) to quantify the effect of the 20 nm SiNPs on the number and degranulation status of mast cells and basophils, which have a role in skin swelling. Analysis of the dorsal side of the ear show that the 20 nm SiNPs had only minimal effects on the number of mast cells/basophils present in the skin (Fig. 4E). Furthermore, while a low percentage of cells were identified as degranulating (<10% for all groups), the number of degranulating mast cells and basophils induced by DNFB were statistically decreased by 20 nm SiNP application (Fig. 4F). The histological findings align with the ear swelling data and suggest that 20 nm SiNPs reduce the effect of DNFB in the CHS response, at 24 hours.

### The 20 nm SiNPs decrease DNFB induced inflammatory cytokine release

**Figure 5:**
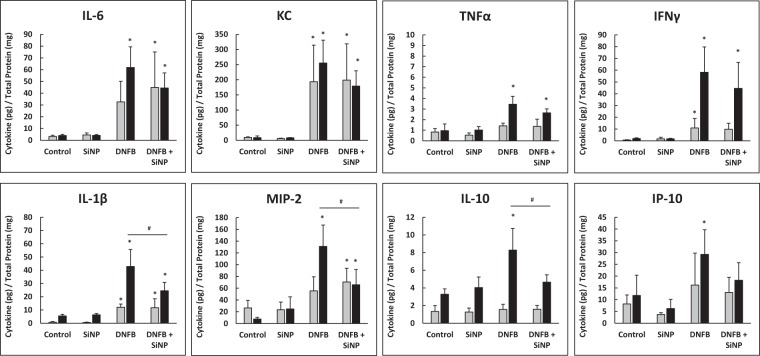
The 20 nm SiNPs decrease DNFB induced cytokine skin expression at 24 hours. The mouse ears were treated with either vehicle, 20 nm SiNPs only, DNFB only, or DNFB + 20 nm SiNPs for 12 or 24 hours. The cytokine expression levels at 12 hours (gray bars) and 24 hours (black bars) are all normalized to the total protein concentration in each skin sample. The graphs represent the mean (SD), n = 4–5, and the * indicates significance compared to control, while the # indicates significance between groups.

To examine the molecular signaling events that led to the decrease in skin swelling, the inflammatory cytokine levels were examined at both 12 and 24 hours, post DNFB application (Fig. 5). DNFB significantly increased the skin expression of interleukin 1 beta (IL-1β), keratinocyte chemoattractant (KC), and interferon gamma (IFNγ) by 12 hours; however, all cytokines displayed significantly higher expression levels after a 24 hour DNFB exposure, compared to control. The 12 hour data suggests that there was no effect of the 20 nm SiNPs on reducing the DNFB induced cytokines, which correlates with the ear swelling time course data displaying no effect on the early phase swelling response. However, at the 24 hour time point, the expression of interleukin 6 (IL-6), IL-1β, KC, macrophage inflammatory protein 2 (MIP-2), and IFNγ were 20–50% lower in the DNFB plus 20 nm SiNP co-treatment group, when compared to DNFB alone. The IL-1β, MIP-2, and interleukin 10 (IL-10) cytokines were the most affected and all reached statistical significance. IL-1β is a general inflammatory cytokine and part of the inflammasome^38^, both KC and MIP-2 are chemoattractants for neutrophils^39,40^, and IL-10 is an anti-inflammatory cytokine^41^. IL-1β is thought to be an essential mediator of CHS, since IL-1 receptor knockout mice display significantly lower allergen induced ear swelling responses^42^. IL-10 is often expressed in the late phase of CHS to reduce the immune response and return the tissue to homeostatic conditions^43^, and while the total expression of IL-10 in the skin is low, the expression pattern may be related to the decreased inflammation that results from SiNP exposure. Altogether, this data indicates that SiNP reduce immune responses in a mechanism independent of IL-10 production. Additionally, there were also decreases in IL-6 and interferon inducible protein 10 (IP-10), although neither reached significance. IL-6 is a general inflammatory cytokine and acute phase protein that can be produced by macrophage or keratinocytes^44^, and IP-10 is produced in response to IFNγ to enhance the recruitment of T cells^45–47^. Since many of these cytokines have downstream effects on immune cell trafficking, the number of immune cell infiltrates per ear were examined next.

### The 20 nm SiNPs decrease DNFB induced immune cell infiltration in the skin

**Figure 6:**
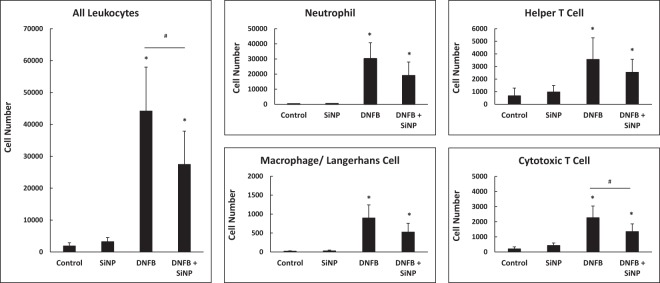
The 20 nm SiNPs decrease DNFB induced leukocyte skin infiltration at 24 hours. The mouse ears were treated with either vehicle, 20 nm SiNPs only, DNFB only, or DNFB + 20 nm SiNPs for 24 hours. The total cell counts per ear were used to obtain the cell number of each of the following immune cells: general leukocytes (CD45+), helper T cells (CD3+, CD4+), cytotoxic T cells (CD3+, CD8+), macrophage/Langerhans cell (F4/80+), and neutrophils (CD11b+, GR-1+). The graphs represent the mean (SD), n = 5–6, and the * indicates significance compared to control, while the # indicates significance between groups.

After a 24 hour treatment with DNFB and 20 nm SiNPs, the whole ears were processed, stained, and analyzed via flow cytometry to assess the number of immune cells per ear. The 20 nm SiNPs had no effect on immune cell infiltration alone; however, they did reduce total leukocyte (CD45+) infiltration induced by DNFB (Fig. 6). A deeper examination of the type of leukocytes with altered skin infiltration patterns indicates that neutrophils (CD11b+, GR-1+), macrophage/Langerhans cells (F4/80+), and helper T cells (CD3+, CD4+) were all reduced by at least 20%, but not significantly. The only individual cell type to display a statically significant reduction in number was the cytotoxic T cell (CD3+, CD8+). Since the 20 nm SiNPs only reduce the late phase CHS swelling response, it is logical that these particles reduce the infiltration of cytotoxic T cells, a late phase mediator of CHS skin swelling^48,49^. However, the SiNPs are unlikely to interact directly with the T cells, since these infiltrates are not present in the early hours of the CHS response. Hence, it is more plausible that the NPs interact with keratinocytes, the most abundant cell type in the epidermis^50^.

### *In vitro* exposure to 20 nm SiNPs decrease DNFB induced HaCaT cytotoxicity and inflammatory cytokine release

**Figure 7:**
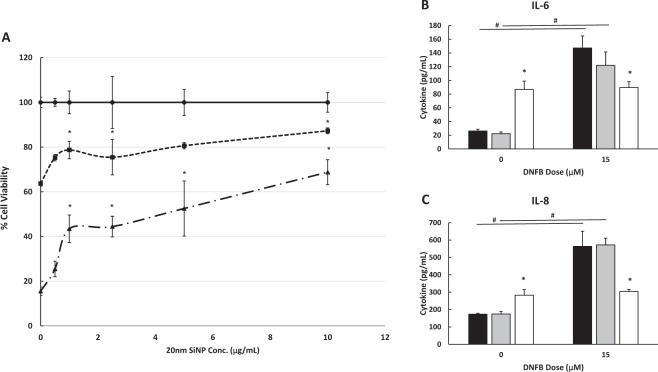
The 20 nm SiNPs decrease DNFB induced cytokine release and cell death in a keratinocyte cell line. The HaCaT keratinocyte cell line was exposed to vehicle (solid line), 15 μM DNFB (short dashed line), or 20 μM DNFB (long dashed line); along with doses of 20 nm SiNPs ranging from 0–10 μg/mL, for 24 hours (**A**). The data are normalized to the respective vehicle treatment to highlight the protective effects when SiNPs are co-administered with DNFB. The IL-6 (**B**) and IL-8 (**C**) cytokine levels in the cell culture media are also presented for select doses of DNFB and SiNPs. SiNP doses of 0 μg/mL (black bars), 0.5 μg/mL (gray bars), and 5 μg/mL (white bars) were analyzed when co-administered with either 0 μM or 15 μM DNFB. The graphs represent the mean (SD), n = 4–5, and the * indicates significance compared to control, while the # indicates significance between groups.

To identify the role that keratinocytes may have in the SiNP induced immunomodulation of skin, HaCaT keratinocytes were cultured with DNFB with and without 20 nm SiNPs. HaCaT cells were cultured in media containing either vehicle or DNFB (15 μM or 20 μM DNFB) in the presence of 0–10 μg/mL 20 nm SiNPs for 24 hours. The low doses of SiNPs (<50 μg/mL) were specifically chosen to minimize NP induced cytotoxicity (Supplementary Fig. 4), and the data presented here was normalized to the respective vehicle (0 μM DNFB) treated samples, for each SiNP dose. Results show that the 20 nm SiNPs have a protective effect on the HaCaTs, by reducing the DNFB induced cytotoxic response at doses above 1 μg/mL (Fig. 7A). In the absence of silica NPs, DNFB reduced cell viability by 36.3% (15 μM DNFB) and by 84.5% (20 μM DNFB), compared to vehicle (0 μM DNFB). Whereas, in the presence of 10 μg/ml silica NPs, DNFB reduced cell viability by only 12.7% (15 μM DNFB) and 31.2% (20 μM DNFB), compared to vehicle (0 μM DNFB). Since a number of immunomodulatory cytokines are known to be produced by keratinocytes, we examined IL-6 and IL-8 (human neutrophil chemoattractant) released by the HaCaT cells exposed to DNFB and 20 nm SiNP (0, 0.5 and 5 ug/mL). Results show (Fig. 7B,C) that IL-6 and IL-8 both increase after 5 μg/mL 20 nm SiNP, but not 0.5 μg/mL of 20 nm SiNP exposure. Interestingly, 15 μM DNFB also increased IL-6 and IL-8 release; however, the 5 μg/mL dose of 20 nm SiNPs significantly reduced the release of both DNFB induced cytokines which is consistent with having a cytotoxic protective effect (Fig. 7A).

Surprisingly, the 400 nm silica microparticles have a similar effect on the DNFB induced HaCaT cytotoxicity (Supplementary Fig. 5), when applied at the same surface area as the 20 nm SiNPs. Unlike the *in vivo* data, the *in vitro* data suggests that there is no size effect, and particles applied at the same surface area can have a similar protective effect on keratinocytes. The difference between *in vitro* and *in vivo* systems is likely due to dosimetry differences between cultured keratinocytes and mouse skin. In culture, the cells can directly interact with the particles of either size; whereas, *in vivo* the stratum corneum barrier likely limits the interaction of the 400 nm silica microparticles with keratinocytes. Studies suggest that NPs < 45 nm may penetrate barrier disrupted skin, but larger particles may not significantly penetrate skin^51^. Our own study examining the skin permeation of fluorescently labeled silica nanoparticles (27.8+/− 3.4 nm) and silica microparticles (557.6 +/− 35.1 nm) indicate that significantly more NPs penetrate mouse ear skin, compared to silica microparticles. Additionally, the level of penetration of silica NPs was increased after DNFB induced skin barrier disruption (Supplementary Fig. 6). It is also important to determine whether the NPs alter the DNFB or proteins in the culture media. For example, the hydrodynamic diameters of both the 20 nm and 400 nm particles were elevated in the cell culture media (162.0 +/− 7.0 nm and 709.2 +/− 19.8 nm, respectively), indicating an interaction with proteins in the culture media. While it is known that NPs bind proteins^52^, there was no significant alteration of media protein concentration or changes in protein binding of DNFB after 24 hours of culture with surface area equivalent doses of the 400 nm silica microparticles, suggesting no indirect effects of the particles on DNFB bioavailability (Supplementary Fig. 7). While this data doesn’t rule out the effect of other cell types *in vivo*, it does suggest a large role of keratinocytes in the NP induced immunomodulation; however, the mechanism by which the silica particles reduce the keratinocyte response to DNFB remains unknown. Future work will characterize transcriptome based changes *in vivo*, with a special emphasis on keratinocytes, to further elucidate the mechanism of action.

## Discussion

The topical application of 20 nm SiNPs decreased the DNFB induced ear swelling response and epidermal hyperplasia, associated with CHS. Based on our previous work^32^, it seemed that decreased mast cell degranulation would have a larger role in SiNP induced skin immunosuppression; however, we observed only minimal mast cell degranulation at 24 hours, even in the DNFB only treatments, that alone do not explain the differences in ear swelling. However, the *in vitro* HaCaT study indicates a direct interaction of SiNPs on keratinocytes that is thought to have a large role in the decreased epidermal hyperplasia and cytokine release observed *in vivo*. The chemokines MIP-2 and KC recruit neutrophils to the skin^39,40^, and significant reductions in MIP-2 led to a moderate decrease in the total number of neutrophils in the skin, at 24 hours (Fig. 6). Neutrophils are known to produce IP-10, and they are necessary for the subsequent recruitment of cytotoxic T cells, part of the late phase swelling response^39,45,53^. At 24 hours, the number of cytotoxic T cells in the skin was significantly reduced (Fig. 6), and this explains the reduction in the late phase swelling response caused by the SiNPs^49^.

There are relatively few studies on the effects of engineered nanomaterials on skin immune responses. This data, suggesting an immunosuppressive effect of SiNPs, is contrary to the literature that demonstrates the immune stimulating potential of SiNPs and their use as adjuvants in vaccine formulations^11–13,54–57^. However, the reason for the disparity is likely due to the high doses of SiNPs (30–300 μg) used in the vaccine experiments, along with the systemic routes of exposure. In another study, SiNPs were observed to size dependently increase dust mite allergen induced atopic dermatitis symptoms, after intradermal injection of 250 μg of particles^58^. Intradermal injections of titanium dioxide nanoparticles were also reported to exacerbate both the sensitization potential and inflammatory response of allergens^59,60^. A more recent study also found that topical application of 250 μg of SiNPs aggregated with dust mite allergen to the ears of mice led to increased risk for atopic reactions^61^. While the particles tested in the previously mentioned studies were negatively charged, positively charged functionalized SiNPs have no known effect on allergic contact dermatitis^32,62^ suggesting that both charge and size effect the immunomodulatory potential of NP in skin. Besides data from our lab, the only topically applied engineered nanomaterials tested that decrease skin immune responses were a silver nanocrystal and a calcium phosphate nanoparticle, which was specially designed to bind nickel ions^63,64^. Here we report that unmodified, amorphous SiNPs decrease allergen induced skin immune responses, which may be related to both the topical route of administration and the lower, less cytotoxic dose.

There is debate whether NPs can penetrate healthy human skin; however, there is data to suggest that NPs can penetrate mouse skin^65–69^, since it has a thinner stratum corneum and a higher hair follicle density, compared to human skin^70^. While mouse skin is a relatively porous barrier, there is evidence that NPs may also penetrate barrier disrupted human skin^71,72^. Since a large portion of the population has inflammatory skin conditions^26^, this represents a potential group at risk for unintended immunomodulation resulting from SiNP exposure. Therefore, it is more relevant to test the toxicity and potential immunomodulation of chronic, low dose exposures to SiNPs in models of inflammatory skin conditions. However, if used in a controlled manner, exposure to low doses of SiNPs could represent a safe and effective way to control symptoms of inflammatory skin conditions, like allergic contact dermatitis. Although, these findings are preliminary, and additional research examining improved NP formulations and efficacy in human models would be required before therapeutic application of these NPs.

Here we have characterized the downstream effects of 20 nm SiNPs in the CHS model, and identified keratinocytes as a potential upstream mediator of SiNP induced immunosuppression. However, the effect is not particle specific, since we observed that a number of small negatively charged NPs display CHS immunosuppression^32^. Due to the wide variety of sizes and surface chemistries of particles that reduce CHS symptoms, the mechanism of action is likely related to a general property of most NPs, like ROS generation^21,73^. For example, cigarette smoke exposure is known to suppress the ear swelling response in a model of CHS, and the mechanism involves the activation of platelet activating factor receptor by oxidized plasma membrane lipids^74^. There are relatively few studies examining the potential protective effects of SiNPs; however, research suggests that these NPs induce keratinocyte proliferation and expression of nuclear factor (erythroid derived 2)-like 2 (Nrf2), a transcription factor for the antioxidant response element, after low dose exposure^75^. Another study found that Nrf2 induced follistatin production enhanced pulmonary epithelial cell survival, after exposure to SiNPs^76^. Since DNFB and other allergens induce high levels of oxidative stress in skin^33,77^, the SiNP induced expression of genes downstream of Nrf2 could have a protective effect on skin. In fact, Nrf2 knockout mice were observed to have increased ear swelling responses after challenge with a chemical sensitizer^78^. Alternatively, a study also found that SiNPs induce autophagy as a pro-survival mechanism, after low dose exposure to a macrophage cell line^79^. Since SiNP induced transcriptional changes in skin represent a potential mechanism of immunosuppression in the CHS model, future studies will examine genome wide transcriptional changes in the skin after treatment with DNFB and SiNPs.

In summary, while amorphous SiNPs are pro-inflammatory and cytotoxic at high doses, we have identified that topically applied, low doses of negatively charged 20 nm SiNPs are immunosuppressive in a DNFB induced model of allergic contact dermatitis. The SiNPs decrease DNFB induced epidermal hyperplasia, skin cytokine expression, and cytotoxic T cell skin infiltration; which ultimately leads to less skin swelling. This immunomodulatory effect should be studied further, before SiNPs are used as transdermal drug delivery agents in patients with inflammatory skin conditions.

## Methods

### Particle Characterization

The 20 nm SiNPs (MEL0010) and 400 nm silica particles (HKE0145) were purchased from the NanoXact line of silica nanospheres at Nanocomposix. The structure of the particles was analyzed by TEM, after spotting the undiluted NP mixtures onto copper grids. The particles were diluted 200x either in water (pH 6.5) or cell culture media and analyzed via the Malvern Zetasizer Nano ZS instrument, to assess hydrodynamic diameter and zeta potential.

### DNFB and SiNP *in vivo* exposure

Mice used in these studies were a fully immunocompetent, hairless C57BL/6 strain, generated and maintained at the University of Rochester. This mouse possesses a gene mutation that causes alopecia after the first follicular maturation, meaning these mice have hair follicles, but they don’t require hair removal. The mice were all males between the ages of 5–6 months, since we have observed age related differences in CHS response. However, there is no observed difference in the CHS response between age matched male and female mice (Supplementary Fig. 8). The mice were kept on a 12 hour light/dark cycle, and they were provided with food and water ad libitum. All mice were sensitized with 30 μL of 0.05% DNFB (Sigma-Aldrich Cat# D1529) on day 0. On day 5, the mouse ears were pre-measured with digital calipers, the mice were housed individually to prevent cross contamination of topical treatments, and the mice were challenged with DNFB and/or SiNPs.

For ear swelling analyses, the mice were treated with 20 μL of a 3:1 acetone (Sigma Aldrich Cat# AGCN20–25M)/water vehicle on the right ear and 20 μL of an aqueous SiNP solution on the left ear. After 30 minutes, both ears were challenged with 20 μL of 0.2% DNFB in a 4:1 acetone/olive oil (Wegman’s Brand Pure Olive Oil) vehicle and the ear swelling was measured at 24 hours, unless otherwise indicated. To ensure that the particles were unable to react with the DNFB before skin exposure, all mice in this study were pretreated with SiNPs 30 minutes prior to DNFB application. This time allows the NP solution to completely dry on the skin, and our data suggests that a dose of 4 μg of 20 nm SiNPs per ear significantly reduces DNFB induced ear swelling when applied up to 1 hour before or 1 hour after DNFB application (Supplementary Fig. 1).

For all other experiments, all mice were treated with 20 μL of a 3:1 acetone/water vehicle on the right ear and 20 μL of a 0.2 μg/μL 20 nm SiNP solution on the left ear. After 30 minutes, one group of mice were treated with 20 μL of a 4:1 acetone/olive oil (Wegman’s Brand Pure Olive Oil) vehicle, and the other group was challenged with 20 μL of 0.2% DNFB. After 24 hours, the mice were euthanized and the ear tissue was processed for histology, cytokine expression, and flow cytometric analysis. All of the *in vivo* experiments were approved by the University Committee on Animal Resources (UCAR#2010-024/100360) at the University of Rochester Medical Center, and all experiments were conducted in accordance with the appropriate regulations and guidelines.

### DNFB and SiNP *in vitro* exposure

The HaCaT cells, an immortal keratinocyte cell line, was used in all *in vitro* assays. The cells were grown in Dulbecco’s Modified Eagle Medium (DMEM) (Gibco Cat# 11965-092) supplemented with 1% penicillin/streptomycin (Gibco Cat# 15140-122) and 10% fetal bovine serum (Gibco Cat# 10082-147). The cells were incubated at 37 °C, with a 5% carbon dioxide atmosphere. The cells were seeded into 96 well plates at a density of 20,000 cells per well, and they were grown until they reached 70% confluency.

For the cytokine analysis, the cells were cultured in clear 96 well plates. For the cell death assay, the cells were cultured in white 96 well plates. The cells were treated with media containing either a 0.1% dimethyl sulfoxide (DMSO) vehicle, 15 μM DNFB, or 20 μM DNFB. For each DNFB treatment, the cells were also treated with 0–10 μg/mL of 20 nm SiNPs (0–176.7 μg/mL for the 400 nm silica particles). After 24 hours, the cell culture media was removed and frozen for future cytokine analysis. The cell viability was measured by adding 100 μL of the CellTiter-Glo (Promega Cat# G7571) reagent to each well. After 15 minutes, the luminescence was measured with a Turner Biosystems Modulus microplate reader. The luminescence data relates to the amount of ATP in each well, which is directly related to the number of viable cells.

### Skin Histology

The ears were fixed in 10% formalin (Electron Microscopy Sciences Cat# 15740-04) for 48 hours. The fixed ears were embedded in paraffin and 5 μm transverse sections were placed on glass slides. Hematoxylin/eosin (Electron Microscopy Sciences Cat# 26030-20/26051-21) staining was used to observe general histology and obtain measures of the epidermal thickness. The 0.1% toluidine blue (MP biomedicals Cat# 152649) stain is a metachromatic stain used for the identification of both mast cells and basophils. A Nikon Eclipse E800 bright field microscope was used to image the skin histology slides, and epidermal thickness measures were obtained in 6 different images per sample, using ImageJ software^80^. All counts and measures were performed with a 20x microscope objective, and the counts or measures from 6–10 fields of view were averaged for each sample. Analysis was only performed on the dorsal sides of the ear, due to the overt edema observed in the ventral sides of ears that made quantification difficult.

### Cytokine Analysis

The whole ears were placed in 300 μL of T-PER extraction buffer (Thermoscientific Cat# 78510) spiked with HALT protease inhibitor cocktail (Thermoscientific Cat# 78410), at a 1:1000 dilution. The samples were mechanically homogenized and incubated on ice for 30 minutes. The samples were centrifuged for 5 minutes at 12,000 RPM to remove the skin debris. A BCA protein assay (Pierce Cat# 23225) was performed, and all cytokine measures were normalized to the total protein content in the ears. A bead based ELISA kit (Millipore Cat# MCYTOMAG-70K) was analyzed on the Bio-Plex 200 system (Bio-Rad) to measure the level of IL-6, IL-1β, KC, MIP-2, TNFα, IL-10, IFNγ, and IP-10. For the analysis of the cell culture media, a similar kit (Millipore Cat# HCYTOMAG-60K) was used to analyze the level of IL-6 and IL-8.

### Flow Cytometry

After euthanasia, the mouse ears were removed, split in half, and processed into single cell suspensions; as previously reported^66^. The cells suspensions were blocked with a 1:10 dilution of anti-mouse CD16/32 Fc blocking reagent (eBioscience Cat# 14-0161-82) for 15 minutes at 4 °C. The samples were washed with PBS, then 100 μL of a 1:200 dilution of each antibody was added to each sample for 30 minutes at 4 °C, including: CD11b (Biolegend Cat# 101243), CD3 (eBioscience Cat# 11-0032-82), CD45 (eBioscience Cat# 58-0451-82), GR-1 (eBioscience Cat# 47-5931-82), F4/80 (Invitrogen Cat# 4323732), CD4 (Biolegen Cat# 100424), CD8 (eBioscience Cat# 61-0081-82) and fixable viability dye (Invitrogen Cat # 4339520). Lastly, the samples were washed with PBS and then fixed with 2.5% formalin. The cells were processed through an 18-color LSRII flow cytometer (BD Biosciences) at a flow rate of 35 μL/minute, and the data was analyzed with FlowJo version 8.8.7 (FlowJo LLC, Ashland, OR). Appropriate compensation controls and fluorescence minus one samples were used for calibrating the machine and gating the samples. All flow cytometry data was converted to total cell counts per ear, using total cell counts obtained by manually counting the cells in each sample with a hemocytometer.

### Statistics

Statistics were run using JMP Pro version 13.2.1 (SAS Institute Inc., Cary, NC). A two-way analysis of variance with post-hoc Tukey tests were used for the histology, cytokine, flow cytometry, and cell culture data. Student’s T-tests were used to analyze the ear swelling data, since all ear swelling data compares only the right (DNFB only) and left (DNFB + SiNP) ear treatments. All data are presented as means ± standard deviation, and p-values < 0.05 were considered significant.

## Supplementary information

Supplementary Information

## Data Availability

The data used in the generation of all tables and figures is available upon request.
