# Supplementary material for: Amorphous silicon dioxide nanoparticles modulate immune responses in a model of allergic contact dermatitis
Source: Sci Rep. 2019 Mar 25;9:5085. doi: 10.1038/s41598-019-41493-7 (PMC6434075; doi:10.1038/s41598-019-41493-7)
Supplement: Supplementary file 1 — Supplementary Information [file 41598_2019_41493_MOESM1_ESM.docx]

**Amorphous silicon dioxide nanoparticles modulate immune responses in a model of allergic contact dermatitis**

Brian C. Palmer^a^, Samreen Jatana^b^, Sarah J. Phelan^a^, Lisa A. DeLouise^a,b,c,*^

^a^Department of Environmental Medicine, University of Rochester Medical Center, New York, USA

^b^Department of Biomedical Engineering, University of Rochester, Rochester, New York, USA.

^c^Department of Dermatology, University of Rochester Medical Center, Rochester, New York, USA.

*Corresponding Author:

Lisa A. DeLouise

University of Rochester Medical Center

School of Medicine and Dentistry

601 Elmwood Avenue, Box 697

Rochester, NY-14642

Tel: (585) 275-1810

E-mail: [Lisa_DeLouise@urmc.rochester.edu](mailto:Lisa_DeLouise@urmc.rochester.edu)

**SUPPLEMENTARY INFORMATION**

**
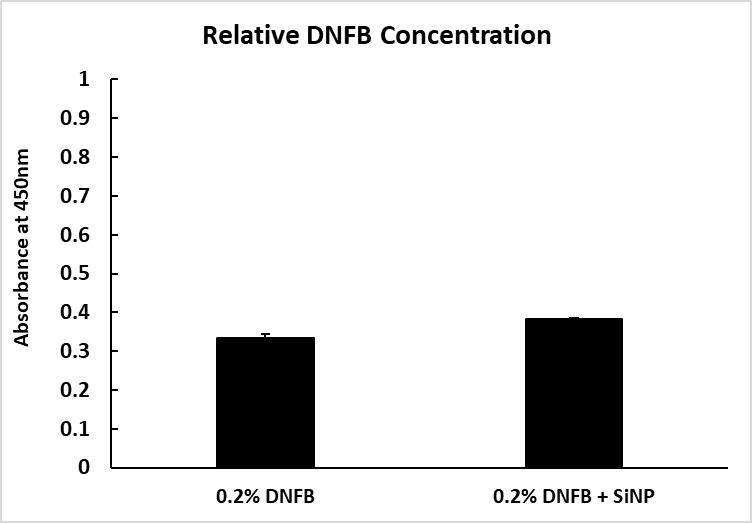
**

Supplementary Figure 1.

Solutions of 0.2% DNFB, dissolved in ethanol, were prepared. The 0.2% DNFB treatment was incubated with only a water vehicle for 24 hours. The 0.2% DNFB + SiNP treatment was incubated with a 5 cm^2^ dose of 400 nm silica particles for 24 hours. After 24 hours, the samples were centrifuged at 14,000 RPM for 20 minutes, to remove the particles. The relative DNFB concentrations were examined via UV-Vis on the NanoDrop 1000 instrument (Thermo Scientific). Since DNFB has a characteristic yellow color, absorbance was measured at 450 nm, and treatment with the silica particles did not reduce the relative absorbance of the DNFB solution, indicating no change in concentration. The graph represents the mean (SD), n = 3.


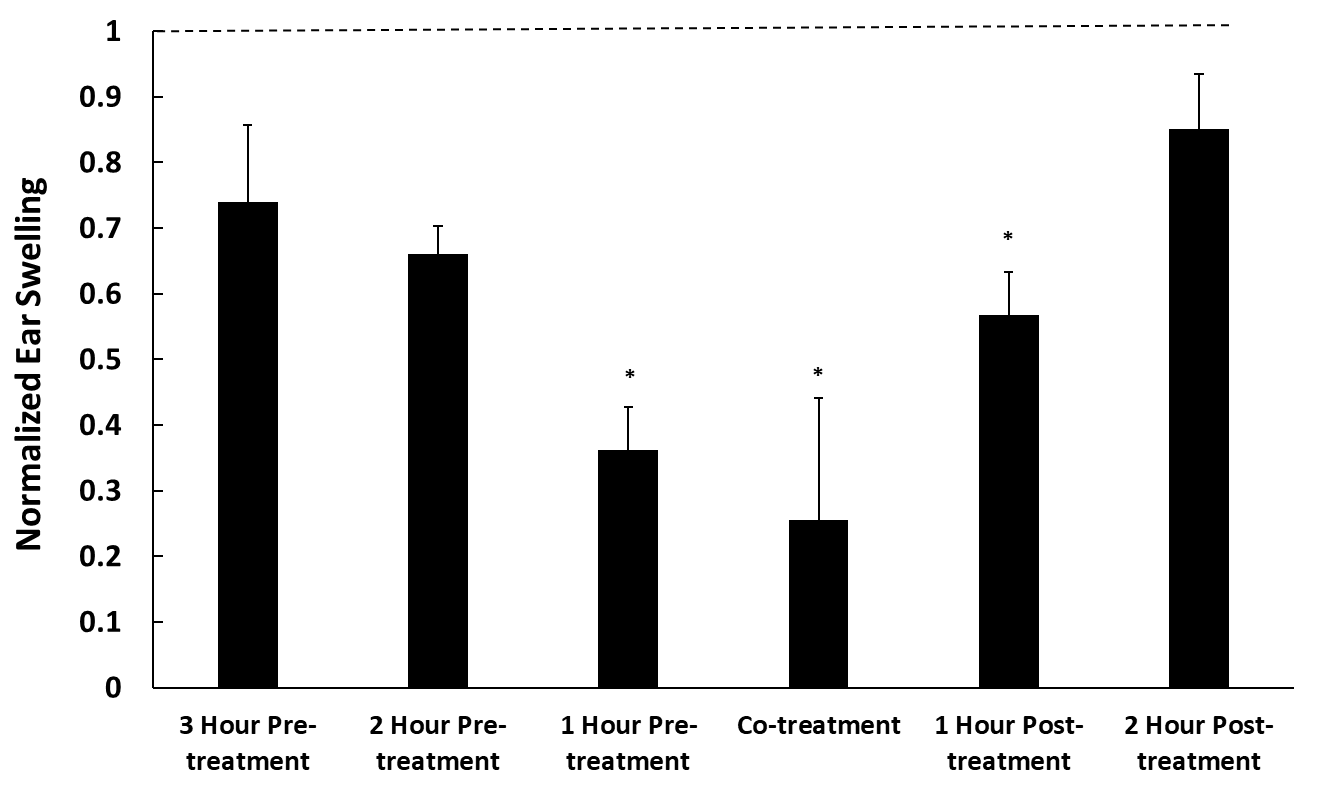


Supplementary Figure 2.

The mice were either treated with 4 μg of 20 nm SiNPs between 1-3 hours prior to 0.2% DNFB challenge, co-administered with DNFB, or they were treated with the SiNPs between 1-2 hours post DNFB challenge. All SiNP and DNFB co-treated ears (bars) are compared to the corresponding DNFB only treated ear (dashed line). Results show statistically significant reductions in ear swelling responses measured 24 hour post challenge when the 20 nm silica NPs are applied 1 hour before DNFB challenge, 1 hour after DNFB challenge or when co-administered. The lack of immunosuppression when particles are applied 2 or 3 hours prior to challenge is likely due to mouse grooming or loss due to cage contact. The lack of immunosuppression when the particles are applied 2 hours post challenge is likely due the onset of the innate immune response that cannot suppressed. Mouse ears were pretreated with silica NP 30 minutes before DNFB challenge for all other studies reported. The graph represents the mean (SD), n = 3, and the * indicates significance compared to the DNFB positive control.


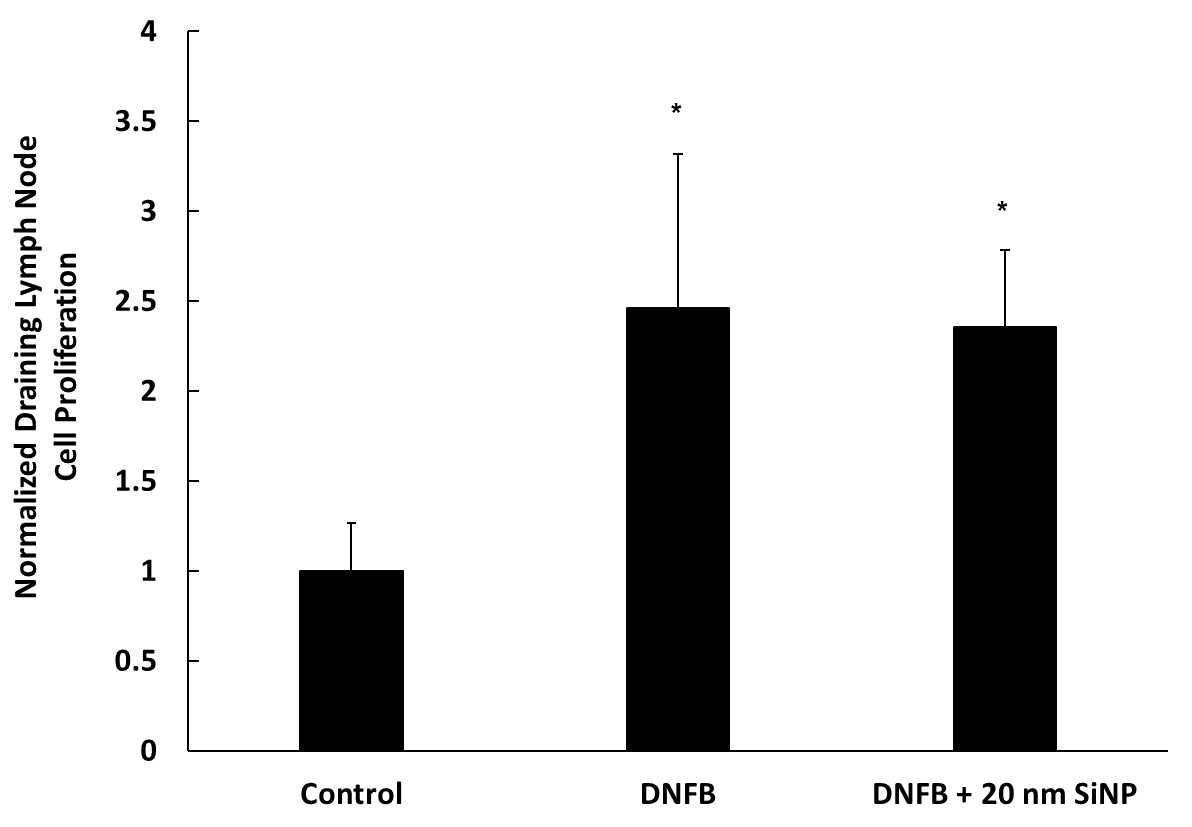


Supplementary Figure 3.

Three sets of mice were sensitized with 30 μL of either vehicle, 0.2% DNFB alone, or 4 μg of 20 nm SiNPs 30 minutes prior to DNFB, on the lower dorsum. After 5 days, the inguinal lymph nodes were removed, and single cell suspensions of each were treated with an ATP reactive luminescent assay, to determine relative amounts of cell proliferation. The data indicates that both DNFB exposed groups have significantly more cell proliferation in the skin draining lymph nodes, compared to control. This local lymph node assay is used to identify contact allergens, and 2-fold increases in lymph node cell proliferation positively correlate with chemical sensitizing potential. The graph represents the mean (SD), n = 6-7, and the * indicates significance compared to control.


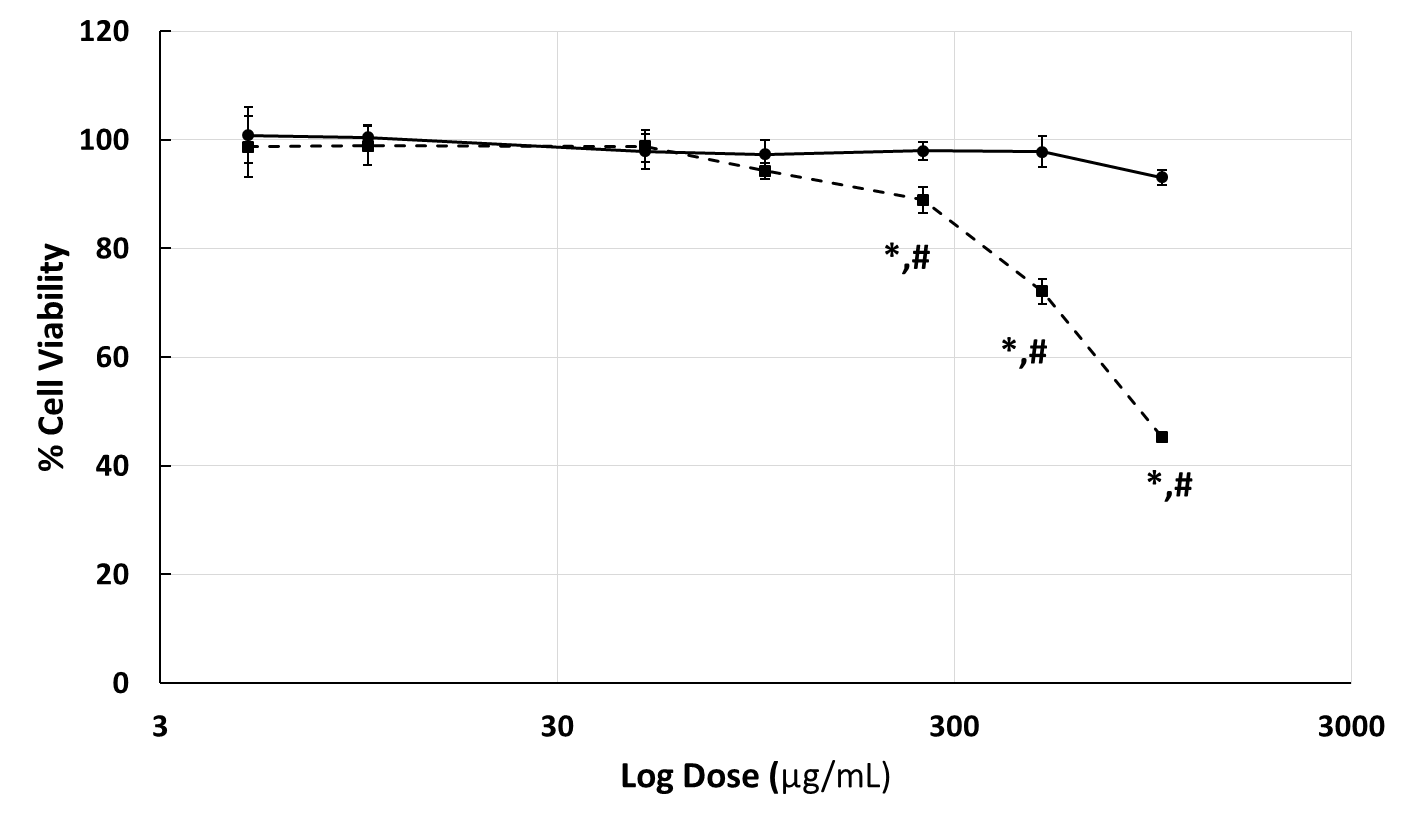


Supplementary Figure 4.

The cell viability data for both the 20 nm (dashed line) and 400 nm (solid line) silica particles, both normalized to vehicle treated HaCaTs. The cells were treated with the silica particles for 24 hours. The data displays a significant reduction in cell viability for 20 nm SiNPs at concentrations ≥250 μg/mL. The graph represents the mean (SD), n = 5, and the * indicates significance compared to control, while the # indicates significance between groups.


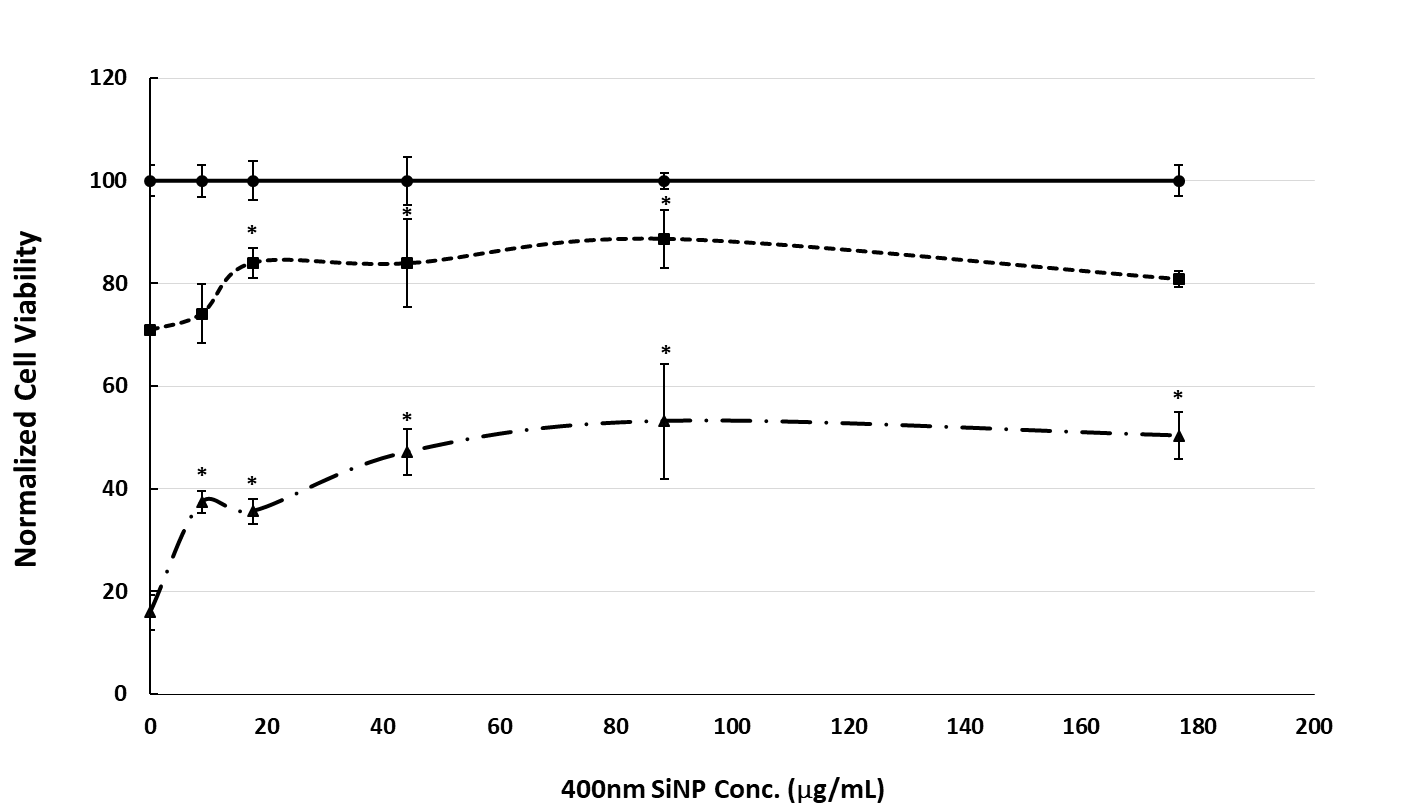


Supplementary Figure 5.

The HaCaT keratinocyte cell line was exposed to DMSO vehicle (solid line), 15 μM DNFB (short dashed line), or 20 μM DNFB (long dashed line); along with doses of 400 nm silica microparticles ranging from 0-176.7 μg/mL (doses are equivalent in surface area to the 20 nm SiNP doses in figure 7), for 24 hours. The data are normalized to the respective vehicle treated sample, for each particles dose, to highlight the protective effects when particles are co-administered with DNFB. The graph represents the mean (SD), n = 4-5, and the * indicates significance compared to control.


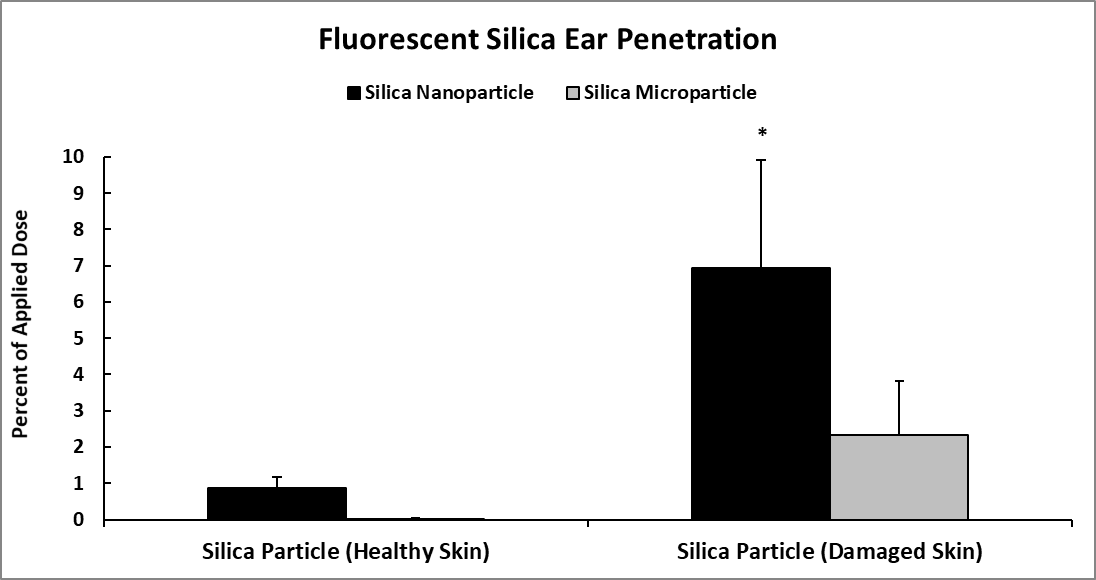


Supplementary Figure 6.

Either untreated dorsal ear skin (healthy skin) or dorsal ear skin post 24 hour challenge with 0.2% DNFB (damaged skin) were treated with either silica nanoparticles (27.8 +/- 3.4 nm) or silica microparticles (557.6 +/- 35.1 nm). The silica particles each contain a red fluorescent dye in the particle core. For each treatment, the ear skin was placed epidermal side up in a Franz diffusion chamber. The lower chamber contained 37^o^ C phosphate buffered saline. Each particle was applied to the epidermis in a 1:1 water/acetone mixture, and the total dose of each particle was 100 μg. After 24 hours, the phosphate buffered saline in the lower chamber was analyzed via a fluorometer (Horiba), to measure the amount of particles that crossed the skin barrier. The fluorescence of each sample was compared to reference samples which contained the whole 100 μg dose, to calculate the percentage of the applied dose that penetrated the ear skin. The graph represents the mean (SD), n = 3. The * indicates significance, compared to every other treatment shown, after two-way ANOVA.


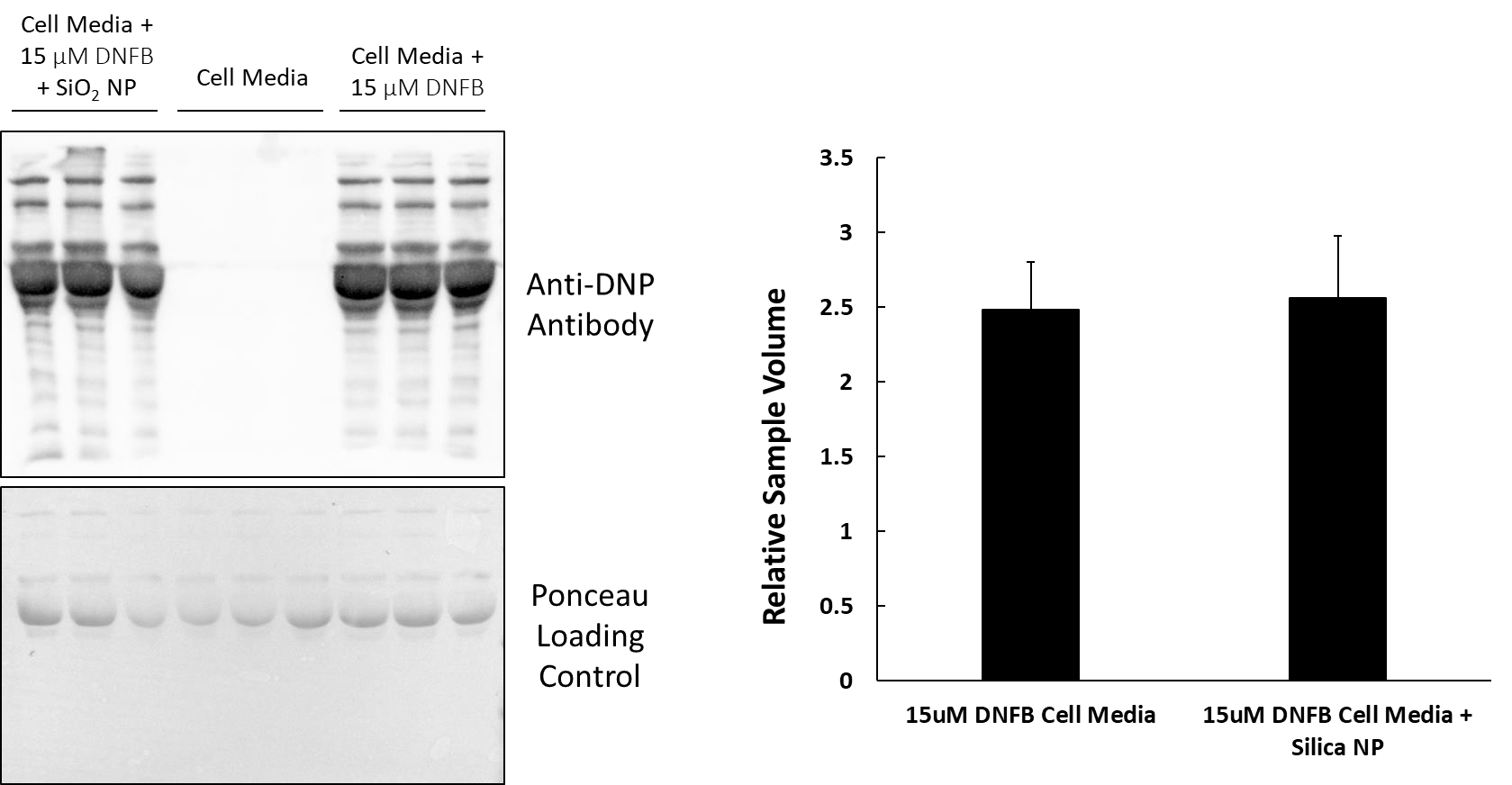


Supplementary Figure 7.

The HaCaT cell culture media was incubated for 24 hours with either vehicle, 15 μM DNFB, or 15 μM DNFB and 176.7 μg/mL of 400 nm silica particles (this dose of particles has a surface area equivalent to the 10 μg/mL dose of 20 nm SiNPs). After 24 hours, the samples were centrifuged at 12,000 RPM for 20 minutes to pellet the 400 nm particles. The protein concentrations of all samples were unchanged by the incubation with the SiNPs, and the DNFB protein adducts were analyzed by Western blotting with an anti-DNP antibody. The uncropped Western blot images were generated by using the auto-exposure feature on the Bio-Rad ChemiDoc MP machine and Image Lab v. 6.0.0 software. The band density was measured across each entire lane, since DNFB may bind all proteins with the appropriate functional groups. The vehicle treatment (cell media without DNFB) samples display the specificity of the antibody for the DNFB adducts, while there was no discernable difference in the level of DNFB adducts in either DNFB treated sample. A ponceau total protein stain was used to control for any difference in the amount of protein loaded in each well. The graph represents the mean (SD), n = 3.


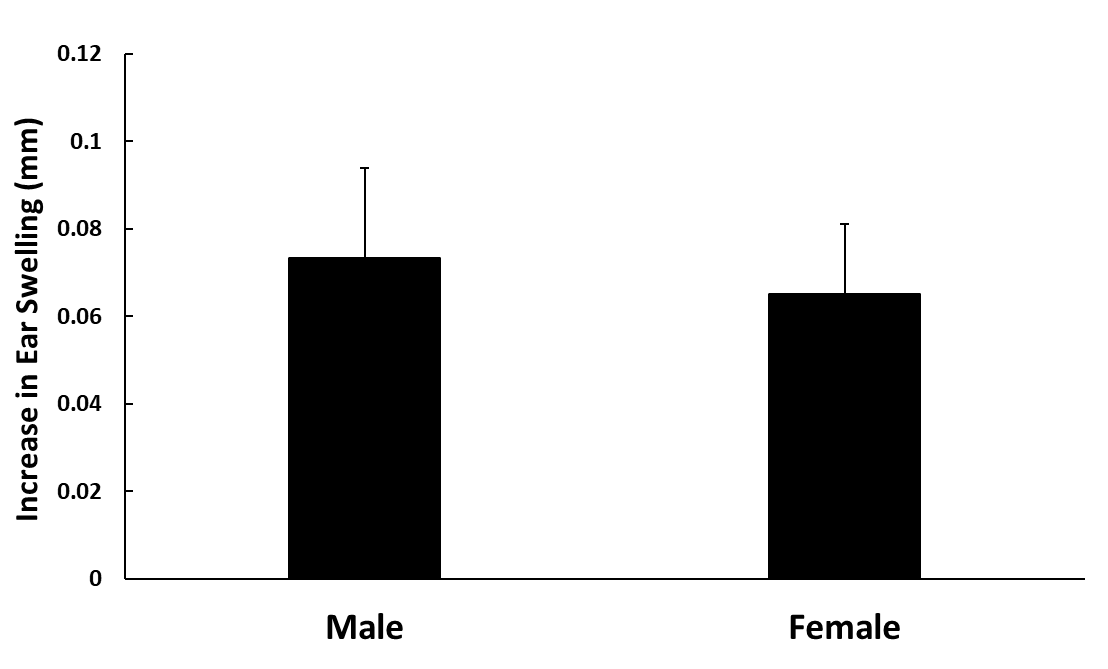


Supplementary Figure 8.

Male and female mice were treated with vehicle on the left ear and 0.2% DNFB on the right ear. After 24 hours, the ear swelling response was measured with digital calipers. There was no difference in the DNFB induced ear swelling response in the male and female mice. The graph represents the mean (SD), n = 3-4.
